# Correction: Do altmetrics correlate with the quality of papers? A large-scale empirical study based on F1000Prime data

**DOI:** 10.1371/journal.pone.0329902

**Published:** 2025-08-06

**Authors:** Lutz Bornmann, Robin Haunschild

In Table 1, the average values for the Twitter counts is incorrect. Please see the correct Table 1 here.

**Table 1:** Average values of the indicators included in this study per publication year.

Publication year	2011	2012	2013
3 years citations, WoS	29.69	29.93	29.95
Citations, WoS	59.04	47.93	33.41
3 years citations, Scopus	33.91	33.38	25.77
Citations, Scopus	58.62	42.51	26.24
F1000 Score	1.91	2.00	2.21
CiteScore	7.10	7.30	7.24
Journal Impact Factor (JIF)	10.67	11.20	11.43
Twitter	4.07	10.29	16.04
Mendeley readers	84.57	75.82	68.97
Altmetric attention score	9.54	15.00	26.83
Number of papers	11,128	11,383	11,172
